# Rodent models of functional hypothalamic amenorrhea: a systematic scoping review

**DOI:** 10.3389/fendo.2025.1456754

**Published:** 2025-06-04

**Authors:** Da-yeong Min, Song-Yi Kim, Ji-Yeun Park, Minseo Kang, Byoung-Soo Kim

**Affiliations:** ^1^ College of Korean Medicine, Daejeon University, Daejeon, Republic of Korea; ^2^ College of Korean Medicine, Gachon University, Seongnam, Republic of Korea

**Keywords:** functional hypothalamic amenorrhea, rodent models, reproductive disorders, primary ovarian insufficiency, polycystic ovary syndrome

## Abstract

**Introduction:**

Functional hypothalamic amenorrhea (FHA) is a complex clinical condition crucial to understand and treat due to its intricate etiology, difficulties in applying standard treatments, and significant long-term health effects. This study aimed to summarize and analyze the current research methodologies and findings from rodent models of FHA to provide insights for future investigations.

**Methods:**

A literature search was conducted on EMBASE and MEDLINE up to September 23, 2022, using predefined search terms to target FHA-related studies in rodent models. This review focused on experimental studies involving rodent models of FHA, including related nonorganic disorders, such as primary ovarian insufficiency (POI) and polycystic ovary syndrome (PCOS). Data were independently collected by researchers, detailing animal models, FHA induction methods, experimental outcomes, and mechanistic exploration, with a synthesis of results comparing FHA with POI and PCOS.

**Results:**

Thirty articles (9 on FHA, 14 on POI, and 7 on PCOS) were analyzed, revealing diverse FHA induction methods, including dietary interventions and exercise, inconsistencies in estrous cycle monitoring, and varied focuses in mechanistic investigation. Some studies have emphasized hypothalamic-pituitary-adrenal axis dysfunction, whereas others have investigated ovarian abnormalities. Comparative analyses of POI and PCOS models identified research gaps and suggested future research directions.

**Conclusions:**

The incorporation of consistent estrous cycle monitoring and biomarker measurements is crucial for the advancement of FHA research. Future studies should comprehensively investigate hormonal changes and explore potential therapeutic targets for ovarian inflammation and androgen involvement.

## 1 Introduction

Functional hypothalamic amenorrhea (FHA) presents a complex clinical challenge, affecting 20–35% of secondary and 3% of primary amenorrhea cases. FHA is defined as a chronic anovulatory condition with no identifiable organic cause (1). Stress from caloric restriction or psychological factors is a significant contributor (2, 3).

Understanding FHA is crucial due to its multifactorial etiology, the challenges in standard treatment, and significant long-term health impacts. Current management is multidisciplinary (medical, dietary, mental health), but many patients struggle with adherence (2) highlighting the need for more effective strategies.

FHA not only causes menstrual dysfunction but also chronic hypoestrogenism, increasing the risk of infertility, bone loss and early osteoporosis, and even cardiovascular issues due to endothelial dysfunction. Additionally, FHA often coexists with anxiety or emotional stress, which further suppresses the hypothalamic–pituitary–gonadal axis and complicates recovery (2–5).

Several rodent studies have tested potential FHA treatments (e.g., agmatine injections (6) or *Milicia excelsa* root extract (7)), but these primarily addressed symptoms rather than underlying mechanisms. A few others induced FHA via specific diets or exercise protocols (8–12); however, each model only captured certain aspects of FHA’s complex pathophysiology. These gaps underscore the need for further rodent studies to elucidate FHA’s underlying biological mechanisms. By leveraging the controlled experimental environment provided by rodent studies, researchers can gain valuable insights into the complex interplay between the factors contributing to FHA development and progression. Such insights are essential for developing more targeted and effective therapeutic interventions to improve the management and long-term outcomes of patients with FHA.

Therefore, this scoping review aimed to identify and summarize all rodent FHA studies, including their induction methods and investigated mechanisms. We also compared FHA models with rodent models of primary ovarian insufficiency (POI) and polycystic ovary syndrome (PCOS) to gain new insights into FHA pathophysiology. This review will contribute to enhancing the understanding of FHA and informing future treatment developments.

## 2 Methods

This scoping review was conducted based on the Preferred Reporting Items for Systematic Review and Meta-Analysis extension for Scoping Reviews checklist (13), with some modifications.

### 2.1 Eligibility criteria

This study focused on FHA in mouse and rat models, setting comprehensive inclusion criteria for the literature search to capture relevant studies, even if they did not explicitly state “FHA” in the title. The inclusion criteria were experimental studies that induced amenorrhea in rats or mice. Subsequently, studies specifically related to FHA were identified in the selected studies. Studies on POI and PCOS were included for comparison with FHA because of their similar nonorganic etiologies. This study aimed to identify the potential areas for further exploration of FHA pathology by examining the biological mechanisms underlying POI and PCOS. We excluded duplicate publications, non-peer-reviewed studies, studies not focused on amenorrhea, non-experimental studies, such as reviews, studies not involving mice or rats, studies not written in English, and studies that were unavailable despite our efforts to obtain the full text.

### 2.2 Information sources and search strategy

The databases used for the literature search were EMBASE and MEDLINE via Embase.com. The search was conducted on September 23, 2022, following a pre-study review and expert consultation to identify key search terms used. The key search terms used to identify studies according to the eligibility criteria are listed in **Supplementary Table S1**.

### 2.3 Selecting sources of evidence

The initial literature search list and data extraction were compiled in Microsoft Office Excel 2010. We established a screening and data extraction strategy to ensure consistency among reviewers. Three researchers (DY Min, MS Kang, and BS Kim) independently screened the literature, and discrepancies were resolved through discussions involving another set of three researchers (JY Park, SY Kim, and BS Kim). The screening process involved sequential steps to exclude studies that did not meet the predefined inclusion criteria. Initially, records that met the preselected exclusion criteria were excluded. Subsequently, a thorough examination of the full text was conducted to confirm whether the literature aligned with this study. Studies not explicitly labeled “FHA” were carefully assessed for inclusion if they met the FHA criteria. In addition, studies in which amenorrhea was not the primary focus, was only considered as a consequence of another disease, or in which amenorrhea was induced with an identifiable organic cause were excluded.

### 2.4 Data charting process and items

Data extraction was independently conducted by two researchers (DY Min and MS Kang), and disagreements were resolved through discussions with other researchers (JY Park, SY Kim, and BS Kim). Information related to the experimental model was extracted, including the animal type, specific species, age of animals, target disease, method of disease induction, and experimental design. Studies that identified related disorders, such as eating disorders and athletic amenorrhea, rather than FHA, were organized as the target disease categories. In addition, the results of the experiments, including animal weight, estrous cycle, ovarian morphology, sex steroid hormones, gonadotropins, and other relevant outcomes, were summarized. Consequently, if efficacious substances were concurrently designed for disease treatment, the extracted information included the substances used, route and duration of administration, and efficacy confirmation time.

### 2.5 Statistical analysis

The characteristics of the studies included in this review were summarized using descriptive statistics. We analyzed the publication time trends of all the included studies. Studies on FHA were categorized into primary or secondary amenorrhea types, and the specific methods used to induce amenorrhea were analyzed and synthesized. Additionally, we verified whether FHA induction was confirmed through reports on the estrous cycle. We analyzed the biomarkers measured in each study and categorized them as serum, organic, total body, and behavioral markers. Additionally, we compared the features mentioned above observed in POI and PCOS rodent studies with those observed in FHA studies.

## 3 Results

### 3.1 Selecting sources of evidence

**Figure 1:**
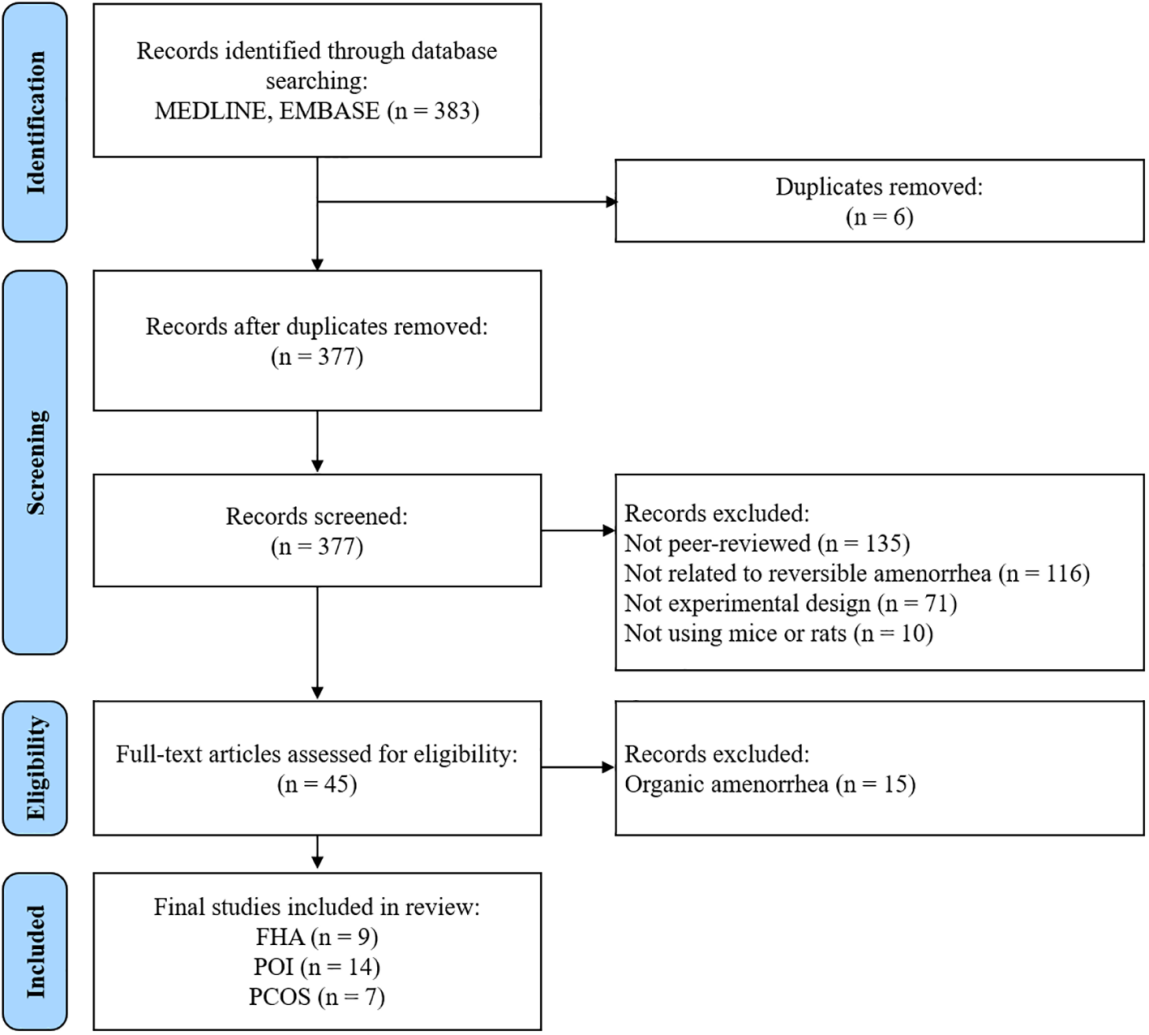
Flow chart of the study selection process. FHA, functional hypothalamic amenorrhea; POI, primary ovarian insufficiency; PCOS, polycystic ovary syndrome.

Overall, 383 studies were identified using the search strategy. After removing six duplicate studies, 377 records remained; we excluded 332 at the abstract stage for not being peer-reviewed, not focusing on amenorrhea, not being experimental, or not using rodent models. Subsequently, 45 full-text articles focusing on diseases with amenorrhea as the primary symptom were examined to meet this study’s objectives. We examined the research design and content to identify the target diseases in each study. The 45 studies were categorized as FHA (n=9) (6–12, 14, 15), POI (n=14) (16–29), PCOS (n=7) (30–36), and organic amenorrhea (n=15) (37–51). The 15 studies classified as “organic amenorrhea” were excluded from the analysis as the condition targeted in these studies could be identified as an organic cause of the disease. Bibliographic details and reasons for excluding these studies are presented in **Supplementary Table S2**. Consequently, 30 studies were included in the final analysis (**Figure 1**). Of these, 30.0% (n = 9) focused on FHA, 46.7% (n = 14) on POI, and 23.3% (n = 7) on PCOS.

### 3.2 Characteristics of the included studies

**Figure 2:**
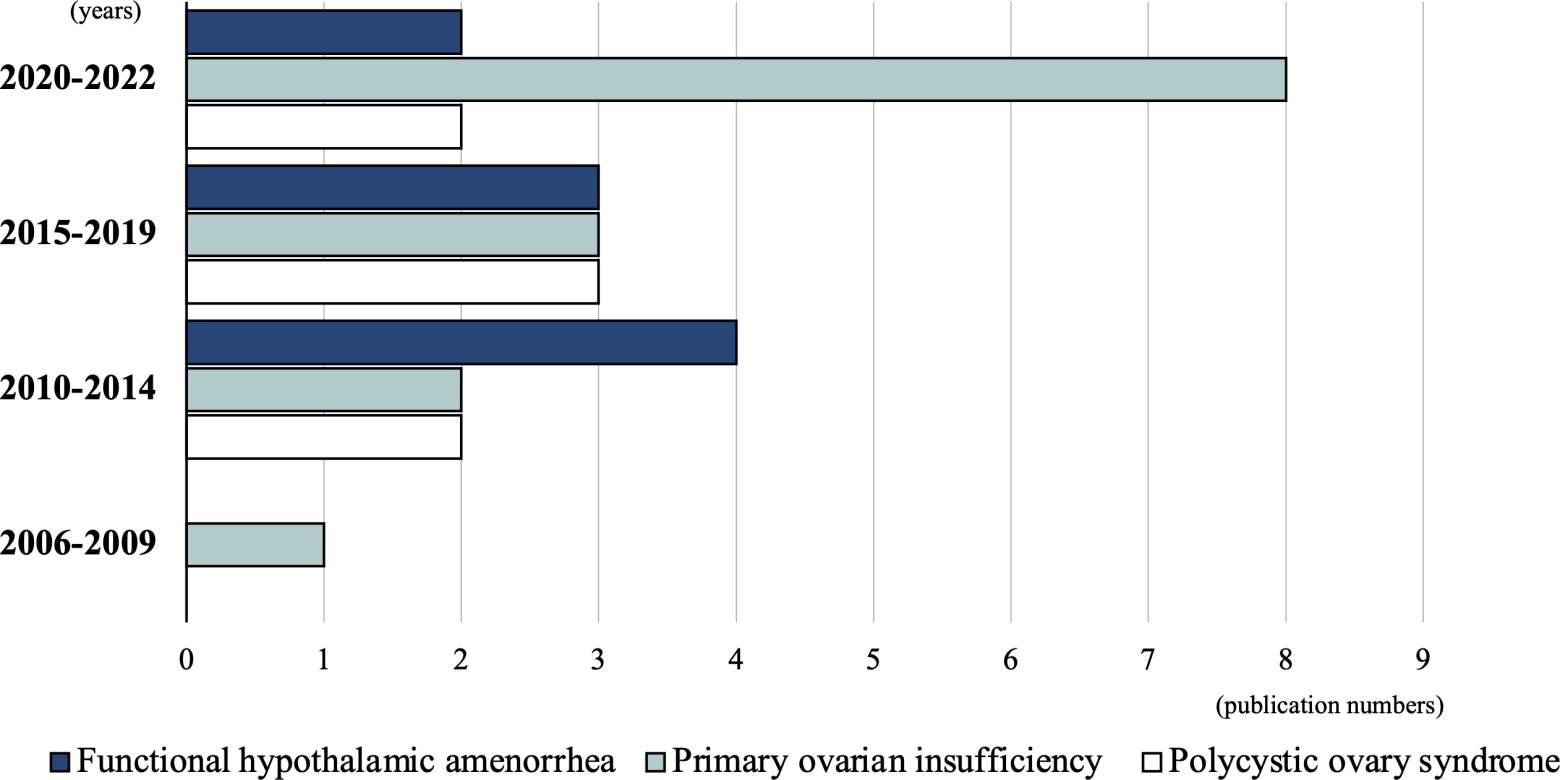
Publication distribution over time.

The 30 studies included in this review were conducted between 2006 and 2022. Before 2009, only one study (3.3%) was conducted, which focused on POI (29). Between 2010 and 2014, eight studies (26.7%) were published: four FHA (11, 12, 14, 15), two POI (27, 28), and two PCOS (35, 36) studies. Between 2015 and 2019, nine studies (30%) were conducted: three FHA (6, 7, 10), three POI (24–26), and three PCOS (32–34) studies. Between September 2020 and 2022, 12 studies (40%) were conducted: eight POI (16–23), two FHA (8, 9), and two PCOS (30, 31) studies. Although the total number of studies is small, an increasing trend is observed, especially in POI-related studies (**Figure 2**).

### 3.3 Characteristics of the FHA rodent studies

#### 3.3.1 Disease induction methods of the FHA rodent studies

**Figure 3:**
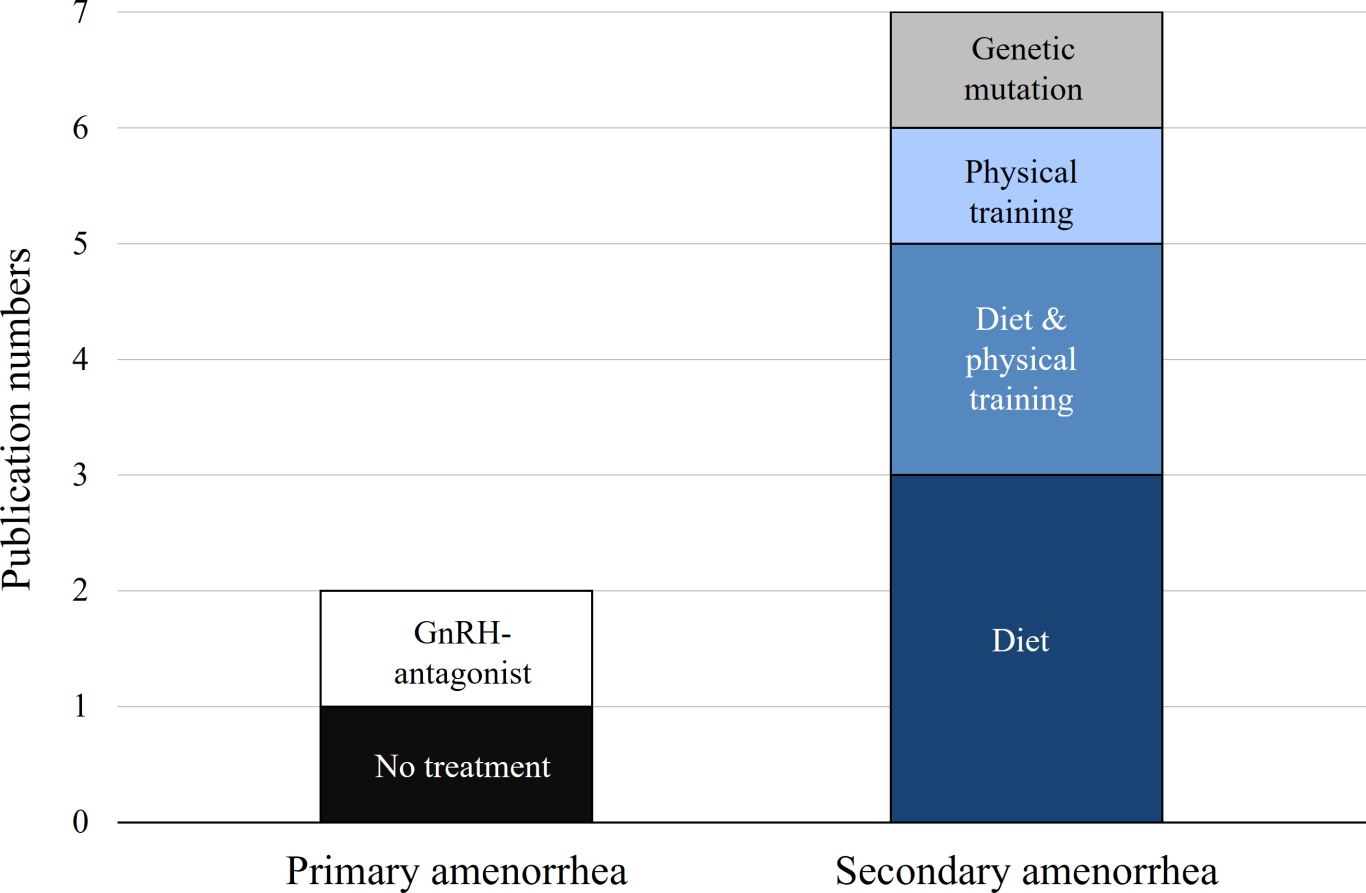
Frequency of disease induction methods by each type of amenorrhea. Primary amenorrhea refers to the failure to initiate estrous cycles after puberty, while secondary amenorrhea refers to the cessation of established estrous cycles. The bars represent the total number of studies involving either primary or secondary amenorrhea. The colors indicate the type of induction method: Light gray = genetic mutation, Light blue = physical training, Medium blue = combined diet and physical training, Dark blue = diet, White = GnRH-antagonist, Black = no treatment.

**Figure 4:**
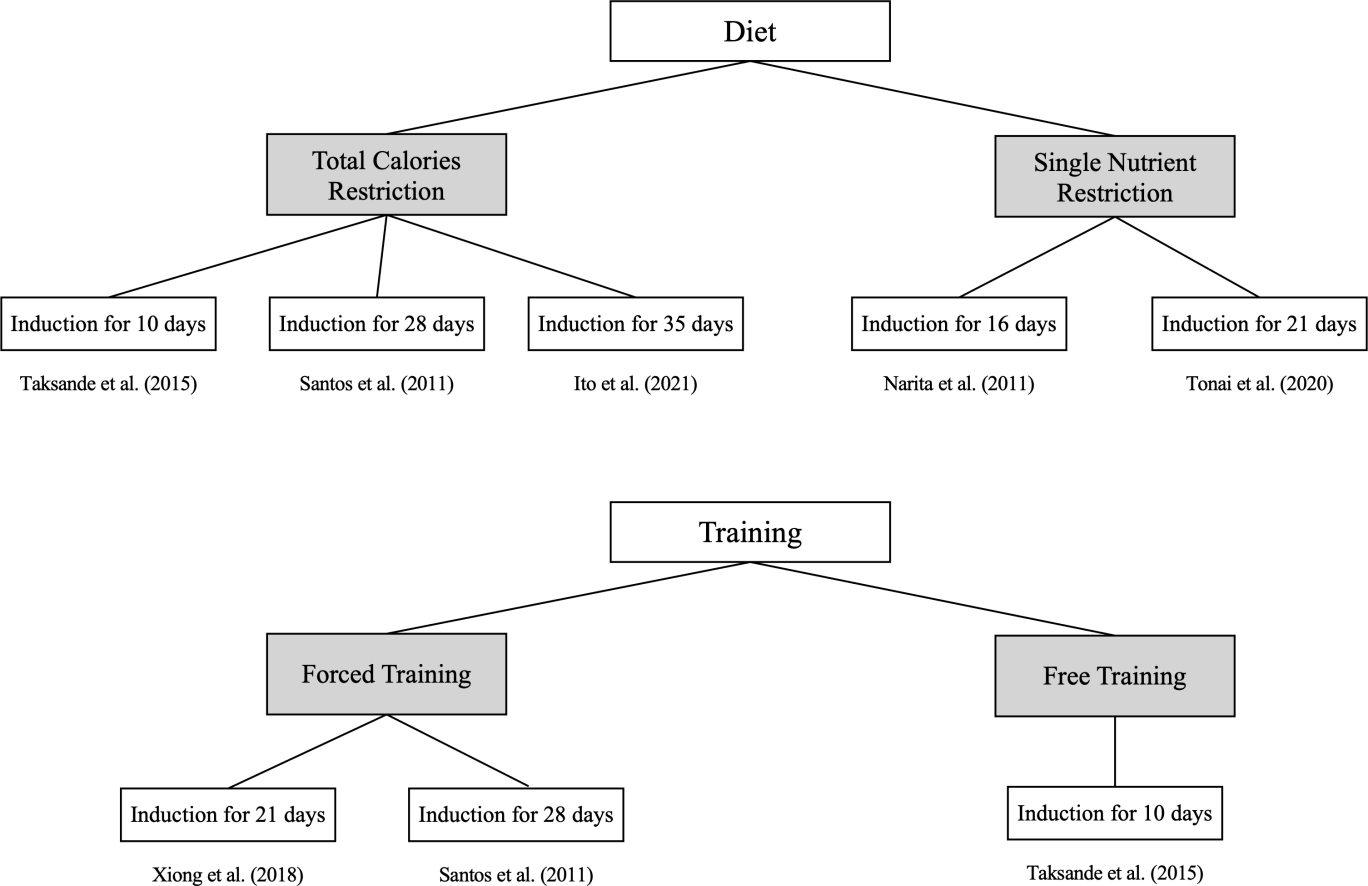
Disease induction duration based on the disease induction method for secondary amenorrhea. Seven studies focused on secondary amenorrhea were classified into diet and training, providing detailed methods and the duration over which the disease was induced.

The details of disease induction in the selected rodent studies are summarized in **Table 1**. Of the nine FHA rodent studies, seven (77.8%) focused on secondary amenorrhea (6, 8–12, 15), while two addressed primary amenorrhea (7, 14). In the primary amenorrhea models, one study (14) used a gonadotropin-releasing hormone (GnRH) antagonist to delay the onset of menarche, whereas another (7) administered an active plant compound (*Milicia excelsa* extract) to accelerate menarche, suggesting stimulation of the hypothalamic–pituitary–ovarian (HPO) axis. For the seven secondary amenorrhea studies, the disease was induced through behavioral or genetic methods: dietary interventions in three studies (42.9%) (8, 9, 12), exercise in one study (14.3%) (10), combined dietary and exercise in two studies (28.6%) (6, 11), and genetic manipulation in one study (14.3%) (15), as illustrated in **Figure 3**. Dietary interventions involve adjusting the overall caloric intake (6, 8, 11) or restricting some nutrients (9, 12). Regarding exercise, some models used forced treadmill running or swimming (10, 11), while others allowed voluntary wheel running (6) (**Figure 4**). One study (15) used genetic knockout of the adrenocorticotropic hormone receptor/melanocortin 2 receptor gene, leading to corticosterone deficiency.

**Table 1:** Details of disease induction of selected rodent studies on functional hypothalamic amenorrhea.

Author (year)	Animal (type)	Age (in days)	Type of amenorrhea induced	Disease induction				
				Target disease	Type of disease induction	Details of disease induction	Disease induction duration (in days)	Estrous cycle report
Ito (2021) (8)	Mouse (C57/B6)	28	Secondary	Food restriction	Diet	Low-calorie diet	35	No
Tonai (2020) (9)	Mouse (C57BL/6JJcl)	21	Secondary	Iron deficiency	Diet	Low Fe diet	21	Yes
Xiong (2018) (10)	Rat (SD)	84	Secondary	Athletic amenorrhea	Physical training	Forced swimming	21	Yes
Mvondo (2017) (7)	Rat (wistar)	45	Primary	Puberty	ND	NA	NA	No
Taksande (2015) (6)	Rat (SD)	60	Secondary	Activity-based anorexia	Diet & physical training	Low-calorie diet and free training	10	Yes
Butler (2013) (14)	Rat (SD)	23	Primary	Puberty	GnRH antagonist	GnRH antagonist injection	18	No
Narita (2011) (12)	Rat (wistar)	42	Secondary	Essential amino acids deficiency	Diet	Single-amino-acid- deficient diet	16	Yes
Dos Santos (2011) (11)	Rat (wistar)	84	Secondary	Food restriction & athletic amenorrhea	Diet & physical training	Low-calorie diet or forced training or a combination of both	28	Yes
Matsuwaki (2010) (15)	Mouse (ND)	ND	Secondary	Functional hypothalamic amenorrhea	Genetic modification	Melanocortin 2 receptor knock-out	NA	Yes

ND, not described. NA, not applicable.

Although each study used a different induction duration, none provided a rationale for their chosen timeframe. The shortest and longest induction periods were 10 and 35 days, respectively (**Table 1**). In Mvondo et al. (7), a therapeutic agent given before menarche advanced its onset, so defining an induction period was not applicable. Matsuwaki et al. (15) used a genetic model causing lifelong dysfunction from birth, making an induction duration impractical to determine.

Six of the nine FHA studies (66.7%) monitored estrous cycle changes, and the reporting formats varied greatly (6, 9–12, 15). Four studies presented estrous cycle data in figure form (9, 11, 12, 15): three showed all four cycle stages, whereas one omitted the metestrus stage (15). Another study labeled the cycle’s peak as ‘proestrus’ instead of distinguishing phases (11).

#### 3.3.2 Measured markers of the FHA rodent studies

We analyzed various biomarkers measured in each rodent FHA study and summarized them in **Table 2**. Although all nine FHA studies induced amenorrhea, the hormones measured varied. Only two studies measured both gonadotropins (follicle-stimulating hormone [FSH] and luteinizing hormone [LH]) (7, 10); others measured only one (9, 15) or neither (6, 8, 14). Estrogen was assessed in four studies (7, 10, 11, 15) – sometimes alongside progesterone or testosterone – whereas the other five did not measure any sex steroids (6, 8, 9, 12, 14).

**Table 2:** Measured markers in FHA rodent studies.

Author (year)	Serum markers				Organic markers		Total body markers	Behavioral markers
	Gonadotropin	Sex steroids	Other hormones	Others	Reproductive system	Other organs		
Ito (2021) (8)	–	–	IGF-1	–	[Uterus] Weight	[Bone] Skeletal morphology	Body weight, body length	Running wheel activity
Tonai (2020) (9)	FSH	-	-	Iron	[Ovary] Weight, follicular morphology, iron, E2, RNA, ATP, Cyp19a1, Ccnd2	[Liver] Iron level	Body weight	Pregnancy rate
Xiong (2018) (10)	FSH, LH	E2, P4, T	–	–	[Ovary] Weight, follicular morphology, mtDNA copy number, ND2, E2, ER, PR	–	Body weight	–
Mvondo (2017) (7)	FSH, LH	E2, P4	-	-	[Uterus] Weight, epithelial height [Vagina] Epithelial height	-	Body weight	-
Taksande (2015) (6)	–	–	Corticosterone	–	–	–	Body weight	Food intake, running wheel activity
Butler (2013) (14)	-	-	-	-	-	[Bone] Growth plate morphology	Body weight	-
Narita (2011) (12)	–	–	IGF-1, leptin, insulin, desacyl ghrelin	Glucose, lipids (TC, TG, NEFA), amino acids	[Ovary] Weight, amino acid	[Liver] Weight, TG	Body weight, total fat weight	Food intake
Dos Santos (2011) (11)	-	E2	Leptin	-	[Ovary] Weight	-	Body weight, chemical composition of the carcass (protein, lipid, perirenal adipose tissue weight etc.)	Food intake
Matsuwaki (2010) (15)	LH	E2	–	–	[Ovary] Follicular morphology [Vagina] Vaginal opening latency	[Brain] CRH, GnRH-containing neuron, kisspeptin	–	Sexual behavior

ATP, Adenosine Triphosphate; Ccnd2, Cyclin D2; CRH, Corticotropin-Releasing Hormone; Cyp19a1, Cytochrome P450 Family 19 Subfamily A Member 1; E2, Estradiol; ER, Estrogen Receptor; FSH, Follicle-Stimulating Hormone; GnRH, Gonadotropin-Releasing Hormone; IGF-1, Insulin-Like Growth Factor; LH, Luteinizing Hormone; mtDNA, Mitochondrial DNA; ND2, NADH Dehydrogenase Subunit 2; NEFA, Non-esterified Fatty Acids; P4, Progesterone; PR, Progesterone Receptor; RNA, Ribonucleic Acid; T, Testosterone; TC, Total Cholesterol; TG, Triglyceride.

The mechanistic focus differed across studies. Tonai et al. (9) induced FHA with an iron-deficient diet and examined ovarian follicular development markers (Adenosine Triphosphate [ATP] levels, FSH receptor, CYP19A1, Cyclin D2). Xiong et al. (10) assessed ovarian mitochondrial function by measuring mtDNA copy number. Taksande et al. (6) and Matsuwaki et al. (15) investigated hypothalamic–pituitary–adrenal (HPA) axis involvement in FHA; notably, Matsuwaki’s genetic model pinpointed an elevated corticotropin-releasing hormone (CRH) effect. Narita et al. (12) measured metabolic hormones (IGF-1, leptin, insulin, ghrelin) to study appetite-related mechanisms. Mvondo et al. (7) monitored gonadotropin and sex steroid levels, as well as reproductive organ development, during delayed puberty. Ito et al. (8) and Butler et al. (14) explored the impact of FHA on bone health, whereas Santos et al. (11) focused on leptin’s role.

We categorized the mechanisms investigated in rodent FHA studies into three groups: HPA axis dysfunction, ovarian dysfunction, and others. Two studies explored the HPA axis (6, 15). Taksande et al. found increased blood cortisol levels owing to excessive exercise-induced anorexia. Matsuwaki et al. created genetically manipulated mice lacking corticosterone secretion. Their results suggested that elevated CRH, not corticosterone, disrupts GnRH secretion. These studies demonstrated the involvement of the HPA axis in FHA. Tonai et al. and Xiong et al. focused on ovarian abnormalities (9, 10). Xiong et al. observed changes in ovarian mitochondrial metabolism, whereas Tonai et al. investigated the alterations in features related to follicular development. Besides, Narita et al. and Ito et al. observed reductions in IGF-1 levels, whereas Santos et al. primarily observed leptin reduction (8, 12).

### 3.4 Comparison between FHA, POI, and PCOS rodent studies

Rodent models of POI and PCOS, which lack structural reproductive system defects and have amenorrhea as a key diagnostic feature, were compared with FHA studies to identify the differences (**Table 3**).

**Table 3:** Summary of experimental characteristics in rodent models of FHA, POI, and PCOS.

Characteristics of animal and physiological changes		FHA (n = 9)	POI (n = 14)	PCOS (n = 7)
Animal type^1^	Rat	6 (66.7%)	2 (14.3%)	5 (71.4%)
	Mouse	3 (33.3%)	12 (85.7%)	2 (28.9%)
Age (in days)^2^	Rat	56.33 ±24.45	56.00 ± 39.60	40.25 ± 21.12^a^
	Mouse	24.50 ± 4.95^b^	46.90 ± 6.94^c^	35.00 ± 9.90
Disease induction duration (in days)^2^	Rat	18.60 ± 6.62^d^	16^e^	20.20 ± 9.26
	Mouse	28.00 ± 9.90^f^	22.00 ± 14.07^g^	47.50 ± 17.68
Estrous cycle report		6 (66.7%)	4 (28.6%)	4 (57.1%)
Gonadotropin^1^	FSH	3 (33.3%)	9 (64.3%)	4 (57.1%)
	LH	3 (33.3%)	2 (14.3%)	5 (71.4%)
Sex steroids^1^	Estrogen	6 (66.7%)	9 (64.3%)	4 (57.1%)
	Progesterone	2 (22.2%)	0	4 (57.1%)
	Testosterone	1 (11.1%)	0	5 (71.4%)
Other hormones		4 (44.4%)	2 (14.3%)	2 (28.6%)
Others^1,3^		2 (22.2%)	4 (28.6%)	4 (57.1%)

^1^Each cell represents the number of studies that apply to it.

^2^Data in the ‘Age’ and ‘Disease induction duration’ row are present as mean ± SEM.

^3^Others includes non-hormonal biomarkers such as inflammatory cytokines, oxidative stress markers, immune modulators, metabolic substrates, and tissue-level indicators. Full details of measured markers are described in **Supplementary Table S4**.

a-c) Studies that did not describe age were excluded; a) Ullah et al. (33), b) Matsuwaki et al. (15), c) Hernández-López et al. (23) and Park et al. (22). d) Mvondo et al. (7) is excluded because their experimental design is distinctive. e) Yuksel et al. (26) is excluded because they induced POI via single administration of drugs. f) Matsuwaki et al. (15) is excluded because they used genetically mutated mice. g) 8 studies are excluded because they induced POI via single administration of drugs.

FHA, Functional Hypothalamic Amenorrhea; FSH, Follicle-Stimulating Hormone; LH, Luteinizing Hormone; PCOS, Polycystic Ovary Syndrome; POI, Primary Ovarian Insufficiency.

The rodent species used across the three disease models varied. Rats were predominantly used in FHA and PCOS studies, whereas mice were more commonly used in POI models. While rats tended to be older than mice, several studies did not report animal age (15, 22, 23, 33), limiting direct comparisons.

The duration of disease induction was generally longer in mouse models than in rat models. However, this comparison is complicated by several POI studies that induced amenorrhea from birth using genetic manipulation (15) or administered high-dose chemotoxic drugs (17, 20, 22, 23, 25–29).

A major distinction among the models lies in the method of disease induction. FHA models typically employed behavioral interventions, such as dietary restriction and/or excessive physical activity. In contrast, POI and PCOS models predominantly used pharmacological methods. Specifically, 12 of the 14 POI studies induced ovarian failure via chemotherapeutic agents (e.g., cyclophosphamide) (16, 18–27, 29), with only two relying on genetic models. Likewise, all seven PCOS studies used hormone-based or enzyme-inhibiting drugs, including testosterone enanthate, letrozole, or dehydroepiandrosterone (30–36) (**Supplementary Table S3**).

Estrous cycle monitoring was reported in 66.7% of FHA studies (6/9 (6, 9–12, 15)), 28.6% of POI (4/14 (19, 22, 28, 29)), and 57.1% of PCOS models (4/7 (31–34)).

An analysis of the measured biomarkers revealed that gonadotropins (FSH or LH) were assessed in 44.4% of FHA studies (4/9 (7, 9, 10, 15)), 64.3% of POI studies (9/14 (16–18, 24, 25, 27–29, 38)), and 71.4% of PCOS studies (5/7 (30–32, 34, 35)).

Similarly, sex steroid hormones (estrogen, progesterone, or testosterone) were measured in 44.4% of FHA studies (4/9 (7, 10, 11, 15)), 64.3% of POI studies (9/14 (16–19, 21, 24, 25, 27, 28)), and 85.7% of PCOS studies (6/7 (30–35)).

Notably, FHA models often included cortisol or appetite-related hormones (leptin, ghrelin, insulin) in their assays. In contrast, PCOS models frequently measured anti-Müllerian hormone (AMH) along with inflammatory cytokines and oxidative stress markers, while POI models also emphasized inflammatory and antioxidant markers. (See **Supplementary Table S4** for the detailed list of markers).

## 4 Discussions

### 4.1 Main findings

Out of 383 screened records, we identified 30 relevant studies, including nine FHA rodent model studies. We found that FHA models employed diverse induction methods but rarely confirmed amenorrhea duration via estrous cycle monitoring. FHA models relied on behavioral triggers (diet/exercise), whereas POI and PCOS models used pharmacological induction. Across all studies, gonadotropins and sex steroids were the primary outcomes measured; uniquely, FHA studies also measured cortisol and appetite-related hormones, whereas POI/PCOS studies focused more on inflammatory and antioxidant markers.

Although FHA, POI, and PCOS are all forms of amenorrhea, the approaches used to establish animal models differed considerably in our review. Unlike POI or PCOS, which are commonly induced through pharmacological interventions, most rodent models of FHA replicate the condition through behavioral manipulations such as caloric restriction or excessive exercise. These approaches mirror the key etiological drivers of FHA observed in humans, specifically energy deficiency due to disordered eating or excessive physical activity (2). Given that FHA is a functional disorder without underlying structural abnormalities, and is primarily driven by psychological and behavioral factors, developing a standardized and reproducible model remains challenging. Quantifying and controlling variables such as stress intensity or exercise load is inherently difficult, and individual variability in response further complicates model consistency. Moreover, since FHA is clinically reversible, it is important that models also demonstrate restoration of the estrous cycle upon removal of stressors. While pharmacological models are not commonly employed for FHA, they may provide a complementary strategy to overcome some of the limitations of behavioral induction. For instance, experimental approaches may include pharmacologically suppressing GnRH activity or artificially elevating stress-related hormones such as CRH or cortisol. Notably, a study (15) included in our analysis demonstrated the potential of targeting the CRH pathway to develop a stress-related FHA model. Nevertheless, pharmacological models often fall short in capturing the multifaceted and systemic nature of FHA. While they may not fully replicate clinical FHA, such models can still serve as valuable tools for testing mechanistic hypotheses or screening targeted therapeutics. Ultimately, selecting an FHA model should be guided by the research objective—whether to closely reflect the clinical condition of FHA or to explore specific mechanistic pathways that are difficult to isolate in human studies. A more integrated approach, linking preclinical and clinical research, will be crucial to filling current gaps and enhancing our understanding of the complex pathophysiology of FHA.

The severity of amenorrhea is an important consideration and can be influenced by the induction method. For instance, an amino-acid–deficient diet delayed estrous cyclicity by day 10 and led to complete cessation by day 14 (12), indicating a progressively severe effect. Tracking the estrous cycle in models is therefore highly informative for gauging FHA severity. Yet only six of nine FHA studies monitored cycle changes (and just four provided graphical cycle data (9, 11, 12, 15)), which made it challenging to compare severity across studies. Monitoring and presentation of all four estrous cycles, including proestrus, estrus, metestrus, and diestrus (e.g., using the clear graph format of Tonai et al. (9)) would greatly aid comparability.

This review categorized the mechanisms of FHA rodent studies into three groups: HPA axis dysfunction, ovarian dysfunction, and others. Some studies observed increased blood cortisol levels owing to excessive exercise-induced anorexia (6, 15), while others focused on ovarian abnormalities, such as changes in mitochondrial metabolism and follicular development (9, 10). Some studies have also observed reductions in IGF-1 and leptin (8, 12).

Notably, most FHA rodent studies observed post-induction changes without delving into mechanisms (Matsuwaki et al. (15), who examined the CRH pathway via genetic manipulation, was a rare exception). This gap is evident in the limited measurement of key HPO-axis hormones in many studies. FHA is fundamentally an HPO-axis disorder – diminished GnRH leads to low FSH/LH, impaired follicular development, and hypoestrogenism (2) – so future FHA models should measure these hormones and be designed to target such mechanistic pathways. More controlled experiments directly examining the HPO-axis dysfunction in FHA will yield deeper insights.

### 4.2 Interpretation

We compared FHA models with POI and PCOS models because all three conditions feature amenorrhea without overt structural abnormalities. Mechanistic insights from POI and PCOS (e.g., ovarian follicle apoptosis in POI or metabolic dysfunction in PCOS) can thus shed light on FHA’s pathophysiology.

In POI (an ovarian failure condition), research focuses on ovary-specific mechanisms like apoptosis, pyroptosis, and mitochondrial dysfunction. Although FHA originates from hypothalamic dysfunction, incorporating ovarian-level analyses — such as follicular apoptosis or mitochondrial activity, as done in POI models — could deepen our understanding of its downstream effects. Similarly, PCOS, particularly the phenotype characterized by polycystic ovarian morphology (PCOM) without hyperandrogenism, shares considerable overlap with FHA. Notably, this PCOM subtype is now considered by some to fall within of the FHA spectrum (52). One common mechanism may involve excessive sympathetic activity, as demonstrated in PCOS models with heightened catecholamine output (53–56). Interestingly, the Taksande model of FHA (6), which combines exercise and caloric restriction, also showed elevated cortisol levels and hyperactivity — suggesting potential involvement of ovarian sympathetic innervation.

Another overlapping pathway involves androgens. Adequate androgen levels are crucial for LH secretion and follicular responsiveness, with peripheral fat contributing substantially to circulating testosterone (57, 58). In the FHA model by Xiong et al., excessive exercise resulted in reduced LH and testosterone levels, indicating that impaired androgen support may play a role in FHA pathophysiology. These observations suggest that energy imbalance in FHA may lead to reduced androgen availability, subsequently affecting gonadotropin secretion and ovarian function.

To build on these mechanistic insights, future FHA studies should go beyond observational descriptions and pursue causal testing through targeted experimental approaches. For example, the role of CRH in inhibiting GnRH secretion — as suggested in models with elevated stress response — can be validated using CRH receptor antagonists or CRHR1 knockdown strategies (59). Likewise, ovarian-specific genetic manipulations, such as FSH receptor or mitochondrial regulator (e.g., PGC-1α) knockouts, may help delineate the downstream effects of energy deficiency and chronic stress (60, 61). These approaches, in conjunction with advanced techniques like RNA sequencing, JC-1 mitochondrial potential assays, and Western blotting of HPO-axis proteins (62–64), will be essential for unraveling FHA’s molecular mechanisms.

Nevertheless, a key limitation of current FHA rodent models is their inability to capture the psychosocial stress component characteristic of human FHA. While energy restriction and physical exertion are well replicated in animals, chronic emotional stress, social context, and individual neuroendocrine resilience — central to clinical FHA — remain challenging to model. This discrepancy restricts translational validity and highlights the importance of integrating multifactorial stress paradigms in future FHA modeling.

Differences in reporting estrous cycle make it difficult to compare FHA severity across models. Accordingly, we recommend that future FHA rodent studies systematically monitor and report all four estrous cycles including proestrus, estrus, metestrus, diestrus via daily vaginal cytology and presenting the data in a clear sequential format (e.g., a timeline of cycle phases) to visualize disruptions. Irregular cycling can be defined as deviations from the typical four–five-day cycle pattern (for instance, prolonged diestrus or erratic phase progression), while amenorrhea (complete cycle cessation) may be operationalized as the absence of estrus for at least two weeks, indicating persistent anestrus. Monitoring and visualization of all estrous cycles will facilitate more accurate comparisons of cycle regularity and amenorrhea severity across models.

We also advocate for measuring diverse biomarker to enhance comparability and mechanistic interpretation across FHA, POI, and PCOS models. The comprehensive hormonal changes related to HPO-axis (GnRH, FSH, LH, estradiol, and progesterone), stress (CRH and cortisol), metabolism (leptin, ghrelin, and insulin), ovarian reserve (AMH), and inflammation/oxidative stress (IL-6, TNF-α, lipid peroxidation markers) will provide a more comprehensive perspective of FHA pathophysiology and facilitate direct comparions with POI and PCOS models, clarifying both shared and unique mechanisms.

### 4.3 Strengths and limitations

This scoping review provides a comprehensive overview of FHA rodent models, summarizing induction methods and mechanistic findings, and emphasizes the need for reporting all estrous cycle and comprehensive biomarker measurement. By comparing FHA with related conditions such as POI and PCOS, we identified key differences in disease modeling approaches, highlighting methodological inconsistencies and gaps in biomarker reporting and cycle monitoring. These insights may help guide future research aimed at refining experimental designs and advancing our understanding of FHA pathophysiology.

However, our study has limitations. This review included only nine FHA studies, which limits the depth of analysis and strength of the conclusions for FHA. Although this study offers a more comprehensive understanding of FHA by comparing and analyzing POI and PCOS studies alongside FHA studies, further foundational research is needed to expand the evidence base on FHA and to support more robust and generalizable conclusions. Moreover, we focused only on rodent models and did not review therapeutic interventions. Future work should examine FHA more comprehensively, including both mechanistic studies and treatment outcomes. Additionally, our findings suggest that androgen activity and ovarian inflammation are underexamined components of FHA pathophysiology, despite their potential links to HPO and HPA axis dysfunction. Given parallels with PCOS and the known impact of stress and metabolism on the reproductive axis, incorporating these factors into future FHA models would enhance their translational relevance.

Based on the gaps identified, we recommend that future FHA rodent studies focus on: (1) reporting changes across all four estrous cycle and conducting comprehensive biomarkers assessments; (2) extending experiment durations to include longer induction and recovery phases; and (3) incorporating multi-axis hormonal and inflammatory profiling (including androgens, CRH, cortisol, cytokines, and ovarian histology). Implementing these steps will improve the comparability of FHA models, clarify their underlying mechanisms, and inform better clinical strategies for managing FHA.

## Data Availability

The original contributions presented in the study are included in the article/**Supplementary Material**. Further inquiries can be directed to the corresponding author.

## References

[1] The Practice Committee of the American Society for Reproductive Medicine . Current evaluation of amenorrhea. Fertil Steril. (2004) 82:33–9. doi: 10.1016/j.fertnstert.2004.07.001 15237040

[2] Gordon CM Ackerman KE Berga SL Kaplan JR Mastorakos G Misra M . Functional hypothalamic amenorrhea: an endocrine society clinical practice guideline. J Clin Endocrinol Metab. (2017) 102:1413–39. doi: 10.1210/jc.2017-00131 28368518

[3] Podfigurna A Meczekalski B . Functional hypothalamic amenorrhea: a stress-based disease. Endocrines. (2021) 2:203–11. doi: 10.3390/endocrines2030020

[4] Indirli R Lanzi V Mantovani G Arosio M Ferrante E . Bone health in functional hypothalamic amenorrhea: What the endocrinologist needs to know. Front Endocrinol. (2022) 13:946695. doi: 10.3389/fendo.2022.946695 PMC9592968 36303862

[5] Shufelt CL Torbati T Dutra E . Hypothalamic amenorrhea and the long-term health consequences. Semin Reprod Med. (2017) 35:256–62. doi: 10.1055/s-0037-1603581 PMC6374026 28658709

[6] Taksande BG Chopde CT Umekar MJ Kotagale NR . Agmatine attenuates hyperactivity and weight loss associated with activity-based anorexia in female rats. Pharmacol Biochem Behav. (2015) 132:136–41. doi: 10.1016/j.pbb.2015.03.005 25782747

[7] Mvondo MA Touomo Sakock AJ Ateba SB Awounfack CF Nanbo Gueyo T Njamen D . Emmenagogue properties of Milicia excelsa (Welw.) C.C. Berg (Moraceae) based, at least in part, on its ability to correlate the activity of the hypothalamic-pituitary axis to that of the ovaries. J Ethnopharmacol. (2017) 206:283–89. doi: 10.1016/j.jep.2017.06.005 28596011

[8] Ito E Sato Y Kobayashi T Nakamura S Kaneko Y Soma T . Food restriction reduces cortical bone mass and serum insulin-like growth factor-1 levels and promotes uterine atrophy in mice. Biochem Biophys Res Commun. (2021) 534:165–71. doi: 10.1016/j.bbrc.2020.11.122 33288195

[9] Tonai S Kawabata A Nakanishi T Lee JY Okamoto A Shimada M . Iron deficiency induces female infertile in order to failure of follicular development in mice. J Reprod Dev. (2020) 66:475–83. doi: 10.1262/jrd.2020-074 PMC7593635 32713881

[10] Xiong RH Wen SL Wang Q Zhou H-Y Feng S . Morphological and molecular variations induce mitochondrial dysfunction as a possible underlying mechanism of athletic amenorrhea. Exp Ther Med. (2018) 15:993–8. doi: 10.3892/etm.2017.5469 PMC5780804 29403550

[11] Dos Santos ZA Da Silva RJ Bacurau RFP Tirapegui J Ribeiro SML . Effect of food restriction and intense physical training on estrous cyclicity and plasma leptin concentrations in rats. J Nutr Sci Vitaminol (Tokyo). (2011) 57:1–8. doi: 10.3177/jnsv.57.1 21512284

[12] Narita K Nagao K Bannai M Ichimaru T Nakano S Murata T . Dietary deficiency of essential amino acids rapidly induces cessation of the rat estrous cycle. PloS One. (2011) 6:e28136. doi: 10.1371/journal.pone.0028136 22132231 PMC3223240

[13] Tricco AC Lillie E Zarin W O’Brien KK Colquhoun H Levac D . PRISMA extension for scoping reviews (PRISMA-ScR): checklist and explanation. Ann Intern Med. (2018) 169:467–73. doi: 10.7326/M18-0850 30178033

[14] Butler TA Yingling VR . The effects of delayed puberty on the growth plate. J Pediatr Orthop. (2013) 33:99–105. doi: 10.1097/BPO.0b013e31826a53f2 23232387 PMC3523281

[15] Matsuwaki T Nishihara M Sato T Yoda T Iwakura Y Chida D . Functional hypothalamic amenorrhea due to increased CRH tone in melanocortin receptor 2-deficient mice. Endocrinology. (2010) 151:5489–96. doi: 10.1210/en.2010-0687 20881239

[16] Liu XH Cai SZ Zhou Y Wang YP Han YJ Wang CL . Ginsenoside Rg1 attenuates premature ovarian failure of D-gal induced POF mice through downregulating p16INK4a and upregulating SIRT1 Expression. Endocr Metab Immune Disord - Drug Targets. (2022) 22:318–27. doi: 10.2174/1871523020666210830164152 34463232

[17] Luo X Xu J Zhao R Qin J Wang X Yan Y . The role of inactivated NF-κB in premature ovarian failure. Am J Pathol. (2022) 192:468–83. doi: 10.1016/j.ajpath.2021.12.005 34971586

[18] Bahrehbar K Khanjarpoor Malakhond M Gholami S . Tracking of human embryonic stem cell-derived mesenchymal stem cells in premature ovarian failure model mice. Biochem Biophys Res Commun. (2021) 577:6–11. doi: 10.1016/j.bbrc.2021.08.063 34487961

[19] Zhou Y Zhou J Xu X Du F Nie M Hu L . Matrigel/umbilical cord-derived mesenchymal stem cells promote granulosa cell proliferation and ovarian vascularization in a mouse model of premature ovarian failure. Stem Cells Dev. (2021) 30:782–96. doi: 10.1089/scd.2021.0005 34030464

[20] Park HS Chugh RM El Andaloussi A Hobeika E Esfandyari S Elsharoud A . Human BM-MSC secretome enhances human granulosa cell proliferation and steroidogenesis and restores ovarian function in primary ovarian insufficiency mouse model. Sci Rep. (2021) 11:4525. doi: 10.1038/s41598-021-84216-7 33633319 PMC7907146

[21] Zhang CR Zhu WN Tao W Lin WQ Cheng C Deng H . Moxibustion against cyclophosphamide-induced premature ovarian failure in rats through inhibiting NLRP3-/Caspase-1-/GSDMD-dependent pyroptosis. Evid Based Complement Alternat Med. (2021) 2021:1–9. doi: 10.1155/2021/8874757 PMC7878072 33613687

[22] Park HS Chugh RM Elsharoud A Ulin M Esfandyari S Aboalsoud A . Safety of intraovarian injection of human mesenchymal stem cells in a premature ovarian insufficiency mouse model. Cell Transplant. (2021) 30:96368972098850. doi: 10.1177/0963689720988502 PMC7894598 33593078

[23] Hernández-López D Geisinger A Trovero MF Santiñaque FF Brauer M Folle GA . Familial primary ovarian insufficiency associated with an SYCE1 point mutation: defective meiosis elucidated in humanized mice. Mol Hum Reprod. (2020) 26:485–97. doi: 10.1093/molehr/gaaa032 32402064

[24] Liu T Wang S Li Q Huang Y Chen C Zheng J . Telocytes as potential targets in a cyclophosphamide-induced animal model of premature ovarian failure. Mol Med Rep. (2016) 14:2415–22. doi: 10.3892/mmr.2016.5540 PMC4991733 27485835

[25] Liu T Wang S Zhang L Guo L Yu Z Chen C . Growth hormone treatment of premature ovarian failure in a mouse model via stimulation of the Notch-1 signaling pathway. Exp Ther Med. (2016) 12:215–21. doi: 10.3892/etm.2016.3326 PMC4906989 27347041

[26] Yuksel A Bildik G Senbabaoglu F Akin N Arvas M Unal F . The magnitude of gonadotoxicity of chemotherapy drugs on ovarian follicles and granulosa cells varies depending upon the category of the drugs and the type of granulosa cells. Hum Reprod. (2015) 30:2926–35. doi: 10.1093/humrep/dev256 26466914

[27] Liu T Qin W Huang Y Zhao Y Wang J . Induction of estrogen-sensitive epithelial cells derived from human-induced pluripotent stem cells to repair ovarian function in a chemotherapy-induced mouse model of premature ovarian failure. DNA Cell Biol. (2013) 32:685–98. doi: 10.1089/dna.2013.2032 PMC3864371 24032550

[28] Ghadami M El-Demerdash E Zhang D Salama SA Binhazim AA Archibong AE . Bone marrow transplantation restores follicular maturation and steroid hormones production in a mouse model for primary ovarian failure. PloS One. (2012) 7:e32462. doi: 10.1371/journal.pone.0032462 22412875 PMC3296713

[29] Altuntas CZ Johnson JM Tuohy VK . Autoimmune targeted disruption of the pituitary-ovarian axis causes premature ovarian failure. J Immunol. (2006) 177:1988–96. doi: 10.4049/jimmunol.177.3.1988 16849513

[30] Moshfegh F Balanejad SZ Shahrokhabady K Attaranzadeh A . Crocus sativus (saffron) petals extract and its active ingredient, anthocyanin improves ovarian dysfunction, regulation of inflammatory genes and antioxidant factors in testosterone-induced PCOS mice. J Ethnopharmacol. (2022) 282:114594. doi: 10.1016/j.jep.2021.114594 34480994

[31] Yang H Lee YH Lee SR Kaya P Hong EJ Lee HW . Traditional medicine (mahuang-tang) improves ovarian dysfunction and the regulation of steroidogenic genes in letrozole-induced pcos rats. J Ethnopharmacol. (2020) 248:112300. doi: 10.1016/j.jep.2019.112300 31606536

[32] Arroyo P Ho BS Sau L Kelley ST Thackray VG . Letrozole treatment of pubertal female mice results in activational effects on reproduction, metabolism and the gut microbiome. PloS One. (2019) 14:e0223274. doi: 10.1371/journal.pone.0223274 31568518 PMC6768472

[33] Ullah A Jahan S Razak S Pirzada M Ullah H Almajwal A . Protective effects of GABA against metabolic and reproductive disturbances in letrozole induced polycystic ovarian syndrome in rats. J Ovarian Res. (2017) 10:62. doi: 10.1186/s13048-017-0359-7 28915843 PMC5603011

[34] Küpeli Akkol E İlhan M Ayşe Demirel M Keleş H Tümen I Süntar İ . Thuja occidentalis L. and its active compound, α-thujone: Promising effects in the treatment of polycystic ovary syndrome without inducing osteoporosis. J Ethnopharmacol. (2015) 168:25–30. doi: 10.1016/j.jep.2015.03.029 25818694

[35] Abramovich D Irusta G Bas D Cataldi NI Parborell F Tesone M . Angiopoietins/TIE2 system and VEGF are involved in ovarian function in a DHEA rat model of polycystic ovary syndrome. Endocrinology. (2012) 153:3446–56. doi: 10.1210/en.2012-1105 22577112

[36] Bas D Abramovich D Hernandez F Tesone M . Altered expression of Bcl-2 and Bax in follicles within dehydroepiandrosterone-induced polycystic ovaries in rats. Cell Biol Int. (2011) 35:423–29. doi: 10.1042/CBI20100542 21062263

[37] Kim MK Yoon JA Yoon SY Park M Lee WS Lyu SW . Human platelet-rich plasma facilitates angiogenesis to restore impaired uterine environments with Asherman’s syndrome for embryo implantation and following pregnancy in mice. Cells. (2022) 11:1549. doi: 10.3390/cells11091549 35563855 PMC9101537

[38] Zhang S Chang Q Li P Tong X Feng Y Hao X . Concentrated small extracellular vesicles from menstrual blood-derived stromal cells improve intrauterine adhesion, a pre-clinical study in a rat model. Nanoscale. (2021) 13:7334–47. doi: 10.1039/D0NR08942G 33889891

[39] Mengue Ngadena YS Owona PE Noubom M Mbock MA MbolangNguegan L Chantal Ngoungouré M . Estrogenic and antioxidant activities of Pterocarpus soyauxii (Fabaceae) heartwood aqueous extract in bilateral oophorectomized Wistar rat. Evid Based Complement Alternat Med. (2021) 2021:1–18. doi: 10.1155/2021/6759000 PMC8497103 34630615

[40] Ouyang X You S Zhang Y Zhang C Zhang G Shao X . Transplantation of human amnion epithelial cells improves endometrial regeneration in rat model of intrauterine adhesions. Stem Cells Dev. (2020) 29:1346–62. doi: 10.1089/scd.2019.0246 32772798

[41] Park M Hong SH Park SH Kim YS Yang SC Kim H-R . Perivascular stem cell-derived cyclophilin A improves uterine environment with Asherman’s syndrome via HIF1α-dependent angiogenesis. Mol Ther. (2020) 28:1818–32. doi: 10.1016/j.ymthe.2020.05.015 PMC7403347 32534604

[42] Shao X Ai G Wang L Qin J Li Y Jiang H . Adipose-derived stem cells transplantation improves endometrial injury repair. Zygote. (2019) 27:367–74. doi: 10.1017/S096719941900042X 31452481

[43] Xia L Meng Q Xi J Han Q Cheng J Shen J . The synergistic effect of electroacupuncture and bone mesenchymal stem cell transplantation on repairing thin endometrial injury in rats. Stem Cell Res Amp Ther. (2019) 10:244. doi: 10.1186/s13287-019-1326-6 PMC6686409 31391117

[44] Sun H Lu J Li B Chen S Xiao X Wang J . Partial regeneration of uterine horns in rats through adipose-derived stem cell sheets†. Biol Reprod. (2018) 99:1057–69. doi: 10.1093/biolre/ioy121 29931041

[45] Zhang L Li Y Guan CY Tian S Lv XD Li J-H . Therapeutic effect of human umbilical cord-derived mesenchymal stem cells on injured rat endometrium during its chronic phase. Stem Cell Res Amp Ther. (2018) 9:36. doi: 10.1186/s13287-018-0777-5 PMC5810045 29433563

[46] Zingue S Michel T Tchatchou J Chantal Beatrice Magne N Winter E Monchot A . Estrogenic effects of Ficus umbellata Vahl. (Moraceae) extracts and their ability to alleviate some menopausal symptoms induced by ovariectomy in Wistar rats. J Ethnopharmacol. (2016) 179:332–44. doi: 10.1016/j.jep.2016.01.004 26771069

[47] Shunmugavel A Khan M Chou PC Singh I . Spinal cord injury induced arrest in estrous cycle of rats is ameliorated by s-nitrosoglutathione: novel therapeutic agent to treat amenorrhea. J Sex Med. (2012) 9:148–58. doi: 10.1111/j.1743-6109.2011.02526.x PMC3809072 22024253

[48] Rossi AGZ Soares JM Jr. Motta ELA Simões MJ Oliveira-Filho RM Haidar MA . Metoclopramide-induced hyperprolactinemia affects mouse endometrial morphology. Gynecol Obstet Invest. (2002) 54:185–90. doi: 10.1159/000068380 12592059

[49] Moro M Inada Y Miyata H Komatsu H Kojima M Tsujii H . Effects of dopamine d2 receptor agonists in a pituitary transplantation-induced hyperprolactinaemia/anovulation model in rats. Clin Exp Pharmacol Physiol. (2001) 28:651–58. doi: 10.1046/j.1440-1681.2001.03495.x 11473532

[50] Kamijima M Ichihara G Kitoh J Tsukamura H Maeda K Yu X . Ovarian toxicity of 2-bromopropane in the non-pregnant female rat. J Occup Health. (1997) 39:144–9. doi: 10.1539/joh.39.144

[51] Lotz W Krause R . Correlation between the effects of neuroleptics on prolactin release, mammary stimulation and the vaginal cycle in rats. J Endocrinol. (1978) 76:507–15. doi: 10.1677/joe.0.0760507 564936

[52] Lauritsen MP Pinborg A Loft A Petersen JH Mikkelsen AL Bjerge MR . Revised criteria for PCOS in WHO G roup II anovulatory infertility–a revival of hypothalamic amenorrhoea? Clin Endocrinol (Oxf). (2015) 82:584–91. doi: 10.1111/cen.12621 25262871

[53] Heider U Pedal I Spanel-Borowski K . Increase in nerve fibers and loss of mast cells in polycystic and postmenopausal ovaries. Fertil Steril. (2001) 75:1141–47. doi: 10.1016/s0015-0282(01)01805-2 11384640

[54] Dissen GA Garcia-Rudaz C Paredes A Mayer C Mayerhofer A Ojeda SR . Excessive ovarian production of nerve growth factor facilitates development of cystic ovarian morphology in mice and is a feature of polycystic ovarian syndrome in humans. Endocrinology. (2009) 150:2906–14. doi: 10.1210/en.2008-1575 PMC2689806 19264868

[55] Lansdown A Rees DA . The sympathetic nervous system in polycystic ovary syndrome: a novel therapeutic target? Clin Endocrinol (Oxf). (2012) 77:791–801. doi: 10.1111/cen.12003 22882204

[56] Wilson JL Chen W Dissen GA Ojeda SR Cowley MA Garcia-Rudaz C . Excess of nerve growth factor in the ovary causes a polycystic ovary-like syndrome in mice, which closely resembles both reproductive and metabolic aspects of the human syndrome. Endocrinology. (2014) 155:4494–506. doi: 10.1210/en.2014-1368 PMC4197978 25211588

[57] Du X Rosenfield RL Qin K . KLF15 is a transcriptional regulator of the human 17β-hydroxysteroid dehydrogenase type 5 gene. A potential link between regulation of testosterone production and fat stores in women. J Clin Endocrinol Metab. (2009) 94:2594–601. doi: 10.1210/jc.2009-0139 PMC2708951 19366843

[58] Zouboulis CC Chen W-C Thornton MJ Qin K Rosenfield R . Sexual hormones in human skin. Horm Metab Res. (2007) 39:85–95. doi: 10.1055/s-2007-961807 17326004

[59] Raftogianni A Roth LC García-González D Bus T Kühne C Monyer H . Deciphering the contributions of CRH receptors in the brain and pituitary to stress-induced inhibition of the reproductive axis. Front Mol Neurosci. (2018) 11:305. doi: 10.3389/fnmol.2018.00305 30214395 PMC6125327

[60] Liu L Hao M Zhang J Chen Z Zhou J Wang C . FSHR-mTOR-HIF1 signaling alleviates mouse follicles from AMPK-induced atresia. Cell Rep. (2023) 42:113158. doi: 10.1016/j.celrep.2023.113158 37733588

[61] Liang H Ward WF . PGC-1α: a key regulator of energy metabolism. Adv Physiol Educ. (2006) 30:145–51. doi: 10.1152/advan.00052.2006 17108241

[62] Qin X Xiao Y Ye C Jia J Liu X Liang H . Pituitary action of E2 in prepubertal grass carp: receptor specificity and signal transduction for luteinizing hormone and follicle-stimulating hormone regulation. Front Endocrinol. (2018) 9:308. doi: 10.3389/fendo.2018.00308 PMC6002485 29937753

[63] Klionsky DJ Abdelmohsen K Abe A Abedin MJ Abeliovich H Acevedo Arozena A . Guidelines for the use and interpretation of assays for monitoring autophagy (3rd edition). Autophagy. (2016) 12:1–222. doi: 10.1080/15548627.2015.1100356 26799652 PMC4835977

[64] Wang Z Gerstein M Snyder M . RNA-Seq: a revolutionary tool for transcriptomics. Nat Rev Genet. (2009) 10:57–63. doi: 10.1038/nrg2484 19015660 PMC2949280

