# Supplementary material for: Rodent models of functional hypothalamic amenorrhea: a systematic scoping review
Source: Front Endocrinol (Lausanne). 2025 Jun 4;16:1456754. doi: 10.3389/fendo.2025.1456754 (PMC12174910; doi:10.3389/fendo.2025.1456754)
Supplement: Supplementary file 1 [file Table1.docx]

**Table S1.** Key search terms.

| Search terms |
| --- |
| (‘mouse:ab,ti’ OR ‘mice:ab,ti’ OR ‘rodent:ab,ti’ OR ‘animal:ab,ti’ OR ‘rats:ab,ti’ OR ‘rat:ab,ti’ OR ‘rodents:ab,ti’) AND (‘amenorrhoea:ab,ti’ OR ‘amenorrhea:ab,ti’) AND [english]/lim |
