# Supplementary material for: Rodent models of functional hypothalamic amenorrhea: a systematic scoping review
Source: Front Endocrinol (Lausanne). 2025 Jun 4;16:1456754. doi: 10.3389/fendo.2025.1456754 (PMC12174910; doi:10.3389/fendo.2025.1456754)
Supplement: Supplementary file 2 [file Table2.docx]

**Table S2.** Details of excluded studies.

| **Literature information** | **Reason of exclusion** |
| --- | --- |
|  |  |
| Kim MK, Yoon JA, Yoon SY, Park M, Lee WS, Lyu SW, Song H. Human Platelet-Rich Plasma Facilitates Angiogenesis to Restore Impaired Uterine Environments with Asherman's Syndrome for Embryo Implantation and Following Pregnancy in Mice. Cells. 2022 May 5;11(9):1549. doi: 10.3390/cells11091549. | Target disease is intrauterine adhesion |
| Zhang S , Chang Q , Li P , Tong X , Feng Y , Hao X , Zhang X , Yuan Z , Tan J . Concentrated small extracellular vesicles from menstrual blood-derived stromal cells improve intrauterine adhesion, a pre-clinical study in a rat model. Nanoscale. 2021 Apr 21;13(15):7334-7347. doi: 10.1039/d0nr08942g. | Target disease is intrauterine adhesion |
| Mengue Ngadena YS, Owona PE, Noubom M, Mbock MA, MbolangNguegan L, Chantal Ngoungouré M, Fifen RN, Bidingha A Goufani R, Kahou Tadah RB, Claude Bilanda D, Kamtchouing P, Dzeufiet Djomeni PD. Estrogenic and Antioxidant Activities of *Pterocarpus soyauxii* (Fabaceae) Heartwood Aqueous Extract in Bilateral Oophorectomized Wistar Rat. Evid Based Complement Alternat Med. 2021 Sep 30;2021:6759000. doi: 10.1155/2021/6759000. | Amenorrhea is caused by ovariectomy |
| Ouyang X, You S, Zhang Y, Zhang C, Zhang G, Shao X, He F, Hu L. Transplantation of Human Amnion Epithelial Cells Improves Endometrial Regeneration in Rat Model of Intrauterine Adhesions. Stem Cells Dev. 2020 Oct 15;29(20):1346-1362. doi: 10.1089/scd.2019.0246. Epub 2020 Sep 2. | Target disease is intrauterine adhesion |
| Park M, Hong SH, Park SH, Kim YS, Yang SC, Kim HR, Noh S, Na S, Lee HK, Lim HJ, Lyu SW, Song H. Perivascular Stem Cell-Derived Cyclophilin A Improves Uterine Environment with Asherman's Syndrome via HIF1α-Dependent Angiogenesis. Mol Ther. 2020 Aug 5;28(8):1818-1832. doi: 10.1016/j.ymthe.2020.05.015. Epub 2020 May 20. | Target disease is intrauterine adhesion |
| Shao X, Ai G, Wang L, Qin J, Li Y, Jiang H, Zhang T, Zhou L, Gao Z, Cheng J, Cheng Z. Adipose-derived stem cells transplantation improves endometrial injury repair. Zygote. 2019 Dec;27(6):367-374. doi: 10.1017/S096719941900042X. Epub 2019 Aug 27. | Target disease is intrauterine adhesion |
| Xia L, Meng Q, Xi J, Han Q, Cheng J, Shen J, Xia Y, Shi L. The synergistic effect of electroacupuncture and bone mesenchymal stem cell transplantation on repairing thin endometrial injury in rats. Stem Cell Res Ther. 2019 Aug 7;10(1):244. doi: 10.1186/s13287-019-1326-6. Erratum in: Stem Cell Res Ther. 2023 Aug 8;14(1):196. | Target disease is intrauterine adhesion |
| Sun H, Lu J, Li B, Chen S, Xiao X, Wang J, Wang J, Wang X. Partial regeneration of uterine horns in rats through adipose-derived stem cell sheets. Biol Reprod. 2018 Nov 1;99(5):1057-1069. doi: 10.1093/biolre/ioy121. | Target disease is intrauterine adhesion |
| Zhang L, Li Y, Guan CY, Tian S, Lv XD, Li JH, Ma X, Xia HF. Therapeutic effect of human umbilical cord-derived mesenchymal stem cells on injured rat endometrium during its chronic phase. Stem Cell Res Ther. 2018 Feb 13;9(1):36. doi: 10.1186/s13287-018-0777-5. | Target disease is intrauterine adhesion |
| Zingue S, Michel T, Tchatchou J, Chantal Beatrice Magne N, Winter E, Monchot A, Awounfack CF, Djiogue S, Clyne C, Fernandez X, Creczynski-Pasa TB, Njamen D. Estrogenic effects of Ficus umbellata Vahl. (Moraceae) extracts and their ability to alleviate some menopausal symptoms induced by ovariectomy in Wistar rats. J Ethnopharmacol. 2016 Feb 17;179:332-44. doi: 10.1016/j.jep.2016.01.004. | Amenorrhea is caused by ovariectomy |
| Shunmugavel A, Khan M, Chou PC, Singh I. Spinal cord injury induced arrest in estrous cycle of rats is ameliorated by S-nitrosoglutathione: novel therapeutic agent to treat amenorrhea. J Sex Med. 2012 Jan;9(1):148-58. doi: 10.1111/j.1743-6109.2011.02526.x. | Amenorrhea is caused by spinal cord injury |
| Rossi AG, Soares JM Jr, Motta EL, Simões MJ, Oliveira-Filho RM, Haidar MA, Rodrigues de Lima G, Baracat EC. Metoclopramide-induced hyperprolactinemia affects mouse endometrial morphology. Gynecol Obstet Invest. 2002;54(4):185-90. doi: 10.1159/000068380. | Target disease is pituitary hyperprolactinemia |
| Moro M, Inada Y, Miyata H, Komatsu H, Kojima M, Tsujii H. Effects of dopamine d2 receptor agonists in a pituitary transplantation-induced hyperprolactinaemia/anovulation model in rats. Clin Exp Pharmacol Physiol. 2001 Aug;28(8):651-8. doi: 10.1046/j.1440-1681.2001.03495.x. | Target disease is pituitary hyperprolactinemia |
| Kamijima M, Ichihara G, Kitoh J, Tsukamura H, Maeda K, Yu X, et al. Ovarian Toxicity of 2‐Bromopropane in the Non‐Pregnant Female Rat. Journal of Occupational Health. 1997 Apr;39(2):144–9. | Amenorrhea is caused by toxic agents |
| Lotz W, Krause R. Correlation between the effects of neuroleptics on prolactin release, mammary stimulation and the vaginal cycle in rats. J Endocrinol. 1978 Mar;76(3):507-15. doi: 10.1677/joe.0.0760507. | Target disease is pituitary hyperprolactinemia |
