# Supplementary material for: Rodent models of functional hypothalamic amenorrhea: a systematic scoping review
Source: Front Endocrinol (Lausanne). 2025 Jun 4;16:1456754. doi: 10.3389/fendo.2025.1456754 (PMC12174910; doi:10.3389/fendo.2025.1456754)
Supplement: Supplementary file 3 [file Table3.docx]

**Table S3.** Details of disease induction of primary ovarian insufficiency and polycystic ovary syndrome rodent studies.

| **Author (year) [Ref]** | **Animal** | **Age (days)** | **Disease induction** | | | |
| --- | --- | --- | --- | --- | --- | --- |
|  |  |  | **Target Disease** | **Details of disease induction** | **Disease induction duration (days)** | **Estrous cycle report** |
| Liu (2022) [16] | Mouse (BALB/c) | 42-56 | POI | D-galactose | 42 | No |
| Luo (2022) [17] | Mouse (C57) | 56 | POI | Cyclophosphamide; genetic mutation | 6 or once | No |
| Bahrehbar (2021) [18] | Mouse (C57/BL6) | 42-56 | POI | Cyclophosphamide | 10 | No |
| Zhou (2021) [19] | Mouse (C57/BL6) | 56 | POI | Cyclophosphamide | 15 | No |
| Park (2021a) [20] | Mouse (C57/BL6) | 42 | POI | Busulphan, cyclophosphamide | Once | No |
| Zhang (2021) [21] | Rat (SD) | 84 | POI | Cyclophosphamide | 16 | No |
| Park (2021b) [22] | Mouse (C57/BL6) | ND | POI | Busulphan, cyclophosphamide | Once | No |
| Hernández-López (2020) [23] | Mouse (B6D2F1) | ND | POI | Genetic mutation | Once | No |
| Liu (2016a) [24] | Mouse (C57/BL6) | 56 | POI | Cyclophosphamide | 21 | No |
| Liu (2016b) [25] | Mouse (C57/BL6) | 49 | POI | Cyclophosphamide | Once | No |
| Yuksel (2015) [26] | Rat (SD) | 28-42 | POI | Cyclophosphamide, cisplatin, gemcitabine | Once | No |
| Liu (2013) [27] | Mouse (C57/BL6) | 42 | POI | Cyclophosphamide | Once | No |
| Ghadami (2012) [28] | Mouse | 42-70 | POI | Genetic mutation | Once | No |
| Altuntas (2006) [29] | Mouse (SWXJ) | 42-56 | POI | Peptide immunization | Once | Yes |
| Moshfegh (2022) [30] | Mouse (NMRI) | 42 | PCOS | Testosterone enanthate | 60 | No |
| Yang (2020) [31] | Rat (SD) | 42 | PCOS | Letrozole | 14 | No |
| Arroyo (2019) [32] | Mouse (C57BL/6N) | 28 | PCOS | Letrozole | 35 | Yes |
| Ullahet (2017) [33] | Rat | ND | PCOS | Letrozole | 36 | No |
| Küpeli (2015) [34] | Rat (SD) | 77 | PCOS | Letrozole | 21 | No |
| Abramovich (2012) [35] | Rat (SD) | 21 | PCOS | Dehydroepiandrosterone | 15 | No |
| Bas (2011) [36] | Rat (SD) | 21-23 | PCOS | Dehydroepiandrosterone | 15 | No |
| ND: not described | | | | | | |
