# Supplementary material for: Rodent models of functional hypothalamic amenorrhea: a systematic scoping review
Source: Front Endocrinol (Lausanne). 2025 Jun 4;16:1456754. doi: 10.3389/fendo.2025.1456754 (PMC12174910; doi:10.3389/fendo.2025.1456754)
Supplement: Supplementary file 4 [file Table4.docx]

**Table S4.** Measured markers in POI and PCOS rodent studies.

| **Author (year) [Ref]** | **Disease** | **Serum markers** | | | |  | **Organic markers** | | **Total body markers** | **Behavioral markers** |
| --- | --- | --- | --- | --- | --- | --- | --- | --- | --- | --- |
|  |  | **Gonadotropin** | **Sex steroids** | **Other hormones** | **Others** |  | **Reproductive system** | **Other organs** |  |  |
| **Moshfegh (2022) [30]** | **PCOS** | FSH, LH | E2, P4, T |  | Antioxidants (GPx, SOD, CAT, GST, GSH), inflammation (IL-6, TNF-α, IL-1β, IL-18, CRP) |  | [Ovary] Follicular morphology, inflammation (IL-6, TNF-α, IL-1β, IL-18 ), hormonal receptors (Fshr, Lhr, Pgr, Esr1), NF-κB Pathway (NF-κB, NF-κB p65, IκB) | - | - | - |
| **Yang (2020) [31]** | **PCOS** | FSH, LH | E2, P4 | - | - |  | [Ovary] Follicular morphology, steroidogenesis (Lhr, Fshr, Pgr, Esr1, Cyp11a1, Cyp19a1, Hsd17b1, aromatase) | - | Body weight | - |
| **Arroyo (2019) [32]** | **PCOS** | LH | T | Insulin | Glucose |  | [Ovary] Follicular morphology | [Gut] Microbiome | Body weight, parametrial fat |  |
| **Ullah (2017) [33]** | **PCOS** | - | E2, P4, T | - | Glucose, blood lipid(TC, TG, HDL, LDL) |  | [Ovary] Follicular morphology, ovary size, total protein, antioxidants (CAT, SOD, POD, GST, GSR, GSR-PX, GSH, T-BARS, ROS) | - | Body weight, body mass, circumference of abdomen and thorax |  |
| **Küpeli Akkol (2015) [34]** | **PCOS** | FSH, LH | E2, P4, T | Leptin | Antioxidants (MDA, SOD, CAT, GPx), glucose, blood lipid(TC, TG, HDL-C, LDL-C) |  | [Ovary] Follicular morphology | [Bone] Osteogenic tissue | - |  |
| **Abramovich (2012) [35]** | **PCOS** | FSH, LH | - | - | - |  | [Ovary] Follicular morphology, angiogenesis (VEGF, FLK1, ANGPT1, ANGPT2, TIE2, vascular area) | - | - |  |
| **Bas (2011) [36]** | **PCOS** | - | P4 | - | - |  | [Ovary] Follicular morphology, apoptosis (Bcl-2, Bax) | - | - |  |
| **Liu (2022) [16]** | **POI** | FSH, LH | E2 | - | Antioxidants (SOD, CAT) |  | [Ovary] Weight, follicular morphology, total protein, cell cycle regulation (p16INK4a, SIRT1) | - | Body weight, hair | Activity, food intake |
| **Luo (2022) [17]** | **POI** | FSH, LH | E2 | AMH | Inflammation (TNF-α, IL-6) |  | [Ovary] Weight, ovary surface area, NF-κB Pathway (p65, p50, IκBα), apoptosis (FasL, Fas, PCNA, Bag1, Bax, Bcl-2), steroidogenesis (Fshr, CYP19A1, CYP17A1), cell cycle regulation (P-AKT, cyclin D1, cyclin D2, FOXO1, FOXO3), ER stress (GRP78, PERK, ATF6, IRE1) | - | Body weight | - |
| **Bahrehbar (2021) [18]** | **POI** | FSH | E2 | - | - |  | [Ovary] Follicular morphology, apoptosis (TUNEL assay) | - | - | - |
| **Zhou (2021) [19]** | **POI** | - | E2 | - | - |  | [Ovary] Follicular morphology, collagen volume, angiogenesis (number of blood vessels, CD31 capillary number, VEGF-A, CD31), Ki67 | - | - | - |
| **Park (2021a) [20]** | **POI** | - | - | - | - |  | [Ovary] Follicular morphology, Ki67 | - | - | - |
| **Zhang (2021) [21]** | **POI** | FSH, LH | E2 | - | Inflammation (IL-1β, IL-18) |  | [Ovary] Ovarian morphology, total protein, pyroptosis (IL-1β, IL-18, cleaved GSDMD, cleaved caspase-1, TXNIP, ASC, NLRP3), NF-κB Pathway (TLR4, NF-κB65, p-NF-κB65, MyD88) | - | - | - |
| **Park (2021b) [22]** | **POI** | - | - | - | - |  | [Ovary] Follicular morphology, apoptosis (TUNEL assay), angiogenesis (CD31), steroidogenesis (Fshr, Cyp19A1)  [Uterus] Ki67, PR, ERα | - | - | Fertility, pup observation |
| **Hernandez-Lopez (2020) [23]** | **POI** | - | - | - | - |  | [Ovary] Follicular morphology, synaptonemal complex (SYCP1, SYCE3, TEX12, gammaH2AX) | - | - | Fertility |
| **Liu (2016a) [24]** | **POI** | FSH | E2 | - | - |  | [Ovary] Weight, follicular morphology, telocyte (CD34/PDGFRα, CD4/PDGFRβ, CD34/C-kit, CD34/vimentin) | - | - | - |
| **Liu (2016b) [25]** | **POI** | FSH | E2 | - | - |  | [Ovary] Weight, follicular morphology, Notch signaling pathway (Notch-1, Cbf-1, Hes-1, CBF-1), cell proliferation factor (Ccnd1), p53 | - | - | - |
| **Yuksel (2015) [26]** | **POI** | - | - | AMH | - |  | [Ovary] Follicular morphology | - | - | - |
| **Liu (2013) [27]** | **POI** | FSH | E2 | - | - |  | [Ovary] Weight, follicular morphology, cell structure (cytokeratin 7, fibronectin, vimentin), ERβ, Ki-67 | - | - | - |
| **Ghadami (2012) [28]** | **POI** | FSH | E2 | - | - |  | [Ovary] Weight, follicular morphology, Fshr  [Uterus] Weight  [Vagina] Weight  [Cervix] Weight | - | Body weight | - |
| **Altuntas (2006) [29]** | **POI** | FSH | - | - | TGF-β superfamily (inhibin-A, activin-A)  immune regulation (IFN-γ, IL-2, IL-5, IL-10) |  | [Ovary] Follicular morphology | Lymph node cell | - | Fertility, pup observation |
| AMH: Anti-Müllerian Hormone; ANGPT1: Angiopoietin-1; ANGPT2: Angiopoietin-2; ATF6: Activating Transcription Factor 6; Bax: Bcl-2-associated X Protein; Bcl-2: B-cell lymphoma 2; Bag1: Bcl-2-associated athanogene 1; CAT: Catalase; CD31: Cluster of Differentiation 31; CD34: Cluster of Differentiation 34; CYP17A1: Cytochrome P450 Family 17 Subfamily A Member 1; CYP19A1: Cytochrome P450 Family 19 Subfamily A Member 1; Cbf-1: C-promoter Binding Factor 1; CRP: C-Reactive Protein; Ccnd1: Cyclin D1; CAT: Catalase; CD31: Cluster of Differentiation 31; CD34: Cluster of Differentiation 34; CAT: Catalase; CD34: Cluster of Differentiation 34; CYP17A1: Cytochrome P450 Family 17 Subfamily A Member 1; CYP19A1: Cytochrome P450 Family 19 Subfamily A Member 1; Cbf-1: C-promoter Binding Factor 1; CRP: C-Reactive Protein; Ccnd1: Cyclin D1; CD4/PDGFRβ: Cluster of Differentiation 4/Platelet-Derived Growth Factor Receptor Beta; CD34/C-kit: Cluster of Differentiation 34/tyrosine-protein kinase Kit; CD34/vimentin: Cluster of Differentiation 34/vimentin; ERα: Estrogen Receptor Alpha; Esr1: Estrogen Receptor 1; FLK1: Fetal Liver Kinase 1; FOXO1: Forkhead Box O1; FOXO3: Forkhead Box O3; FSH: Follicle-Stimulating Hormone; Fas: Tumor Necrosis Factor Receptor Superfamily Member 6; FasL: Fas Ligand; Fshr: Follicle-Stimulating Hormone Receptor; GPx: Glutathione Peroxidase; GSDMD: Gasdermin D; GST: Glutathione S-Transferase; GRP78: Glucose-Regulated Protein 78; GSH: Glutathione; Hes-1: Hairy and Enhancer of Split-1; Hsd17b1: Hydroxysteroid 17-beta Dehydrogenase 1; IFN-γ: Interferon-gamma; IRE1: Inositol-Requiring Enzyme 1; IL-1β: Interleukin-1 Beta; IL-10: Interleukin-10; IL-18: Interleukin-18; IL-2: Interleukin-2; IL-5: Interleukin-5; IL-6: Interleukin-6; IκB: Inhibitor of Nuclear Factor Kappa-B; IκBα: Inhibitor of Nuclear Factor Kappa-B Alpha; Ki-67: Marker of Proliferation Ki-67; Lhr: Luteinizing Hormone Receptor; LDL: Low-Density Lipoprotein; MDA: Malondialdehyde; MyD88: Myeloid Differentiation Primary Response 88; NF-κB: Nuclear Factor Kappa-light-chain-enhancer of Activated B Cells; NF-κB p65: Nuclear Factor Kappa-B Subunit 65; NLRP3: NLR Family Pyrin Domain Containing 3; Notch-1: Neurogenic Locus Notch Homolog Protein 1; PCNA: Proliferating Cell Nuclear Antigen; P-AKT: Phosphorylated Protein Kinase B; Pgr: Progesterone Receptor; PERK: Protein Kinase R-like Endoplasmic Reticulum Kinase; PDGFRα: Platelet-Derived Growth Factor Receptor Alpha; PDGFRβ: Platelet-Derived Growth Factor Receptor Beta; PR: Progesterone Receptor; ROS: Reactive Oxygen Species; SIRT1: Sirtuin 1; SOD: Superoxide Dismutase; SYCE3: Synaptonemal Complex Central Element Protein 3; SYCP1: Synaptonemal Complex Protein 1; T: Testosterone; T-BARS: Thiobarbituric Acid Reactive Substances; TC: Total Cholesterol; TGF-β: Transforming Growth Factor Beta; TEX12: Testis Expressed 12; TNF-α: Tumor Necrosis Factor Alpha; TLR4: Toll-like Receptor 4; TG: Triglyceride; TNF-α: Tumor Necrosis Factor Alpha; TXNIP: Thioredoxin Interacting Protein; VEGF: Vascular Endothelial Growth Factor; gammaH2AX: H2A.X Variant Histone; | | | | | | | | | | |
